# Lipingshu capsule improves atherosclerosis associated with lipid regulation and inflammation inhibition in apolipoprotein E–deficient mice

**DOI:** 10.1186/s12944-018-0823-4

**Published:** 2018-07-31

**Authors:** Jiqu Xu, Congcong Ma, Meng Chen, Shuang Rong, Hui Gao, Zumeng Xia, Fenghong Huang

**Affiliations:** 1 Department of Nutriology, Oil Crops Research Institute, Chinese Academy of Agricultural Sciences, 2 Xudong Second Road, Wuhan, 430062 People’s Republic of China; 2 Hubei Key Laboratory of Lipid Chemistry and Nutrition, Oil Crops Research Institute, Chinese Academy of Agricultural Sciences, 2 Xudong Second Road, Wuhan, 430062 People’s Republic of China; 3 Key Laboratory of Oilseeds processing, Ministry of Agriculture, 2 Xudong Second Road, Wuhan, 430062 People’s Republic of China; 4 Department of Nutrition and Food Hygiene, School of Public Health, Medical College, Wuhan University of Science and Technology, No. 2, Huangjiahu Road, Wuhan, 430065 China; 5 Department of Nutrition and Food HygieneSchool of Public Health, Tongji Medical College, Huazhong University of Science and Technology, 13 Hangkong Road, Wuhan, 430030 People’s Republic of China; 6 Functional Oil Laboratory Associated by Oil Crops Research Institute, Chinese Academy of Agricultural Sciences and Infinite (China) Co., LTD., 66 Jianzhong Road, Guangzhou, 510665 People’s Republic of China

**Keywords:** Lipingshu, Capsule, Atherosclerosis, Lipid profile, Inflammation

## Abstract

**Background:**

Atherosclerosis (AS) is mainly responsible for cardiovascular diseases. The present study investigated whether Lipingshu capsule (LPS), whose ingredients are present in health food stores, has beneficial effect on AS.

**Methods:**

C57BL/6 J mice were given a low fat rodent diet and assigned as control group (CON). ApoE^−/−^ mice were placed on high fat rodent diet and randomly separated into high fat diet (HFD) group and HFD + LPS group whose animals were given 0.9 g/kg.BW LPS daily for 10 weeks. Atherosclerotic lesions in aorta and aortic root were evaluated. Serum lipids and multiple cytokine were measured.

**Results:**

ApoE^−/−^ mice fed with high fat diet had serious aortic lesions, whereas LPS markedly decreased plaque area of the total aorta and of the aortic root. LPS recovered the serum lipid profiles by substantially reducing TC, LDL-C, TG and Ox-LDL contents. Multi-cytokine analysis revealed greater serum levels of IL-1α, IL-1β, IL-6, IFN-γ, GMCSF, RANTES and TNF-α induced by high fat diet slumped with LPS treatment.

**Conclusion:**

LPS reduces atherosclerotic lesions and thus alleviates AS by lipid profile modulation and inflammation inhibition.

## Background

Cardiovascular diseases (CVD), representing 31% of all global deaths, are the first cause of death worldwide according to updated statistics (source: http://www.who.int/mediacentre/factsheets/fs317/en/ accessed Updated May 2017). Atherosclerosis (AS) is the lesion central to CVD including sudden cardiac death, myocardial infarction, unstable angina, peripheral thromboses and stroke. It has been identified as a complex disease process resulting from the interaction between lipid perturbations and inflammation [1].

Dyslipidemia and inflammation are major mechanisms involved in the pathophysiology of atherosclerosis. Hyperlipidemia is characterized serologically by increased plasma triglyceride (TG), total cholesterol (TC) and low density lipoprotein cholesterol (LDL-C) levels [2]. Hypercholesterolemia is unique in being sufficient to result in atherosclerotic damage, even in the absence of other cardiovascular risk factors [3]. Elevated circulating LDL-C level is recognized as the major risk factor for AS, as supported by clinical evidence showing decreased atherosclerotic disease events when LDL-C was therapeutically lowered. The LDL oxidative modification (oxLDL) is crucial in atherogenesis [3–5] since oxLDL promotes AS progression directly by many mechanisms [3, 6–8] and served as a biomarker of CVD [9]. Evidence suggests that hypertriglyceridemia may also pose a significant risk for CVD [10, 11]. AS is likewise a chronic a chronic inflammatory process. Many risk factors such as dyslipidemia, hypertension and obesity trigger multiple inflammatory reactions, which lead to monocytes recruitment and foam cells formation in AS lesions [12]. The interactions between disordered lipid metabolism and inflammatory processes aggravate the development of AS [1].

Traditional Chinese Medicine (TCM) has been widely used for thousands of years in China, and now it is also deemed to treat various diseases or promote health. Some TCM formulas have been reported to possess antiatherosclerotic properties [13, 14]. As a potential ingredient of functional food, there are direct evidences for the effects of milk-derived bioactive peptides against atherosclerosis by many mechanisms including anti-inflammatory and hypolipidemic activities [15–17]. Lipingshu capsule (LPS) is an innovative formula and its major ingredients include Gastrodia elata Bl.(Tianma), *Eucommia ulmoides* Oliver (Duzhong), Apocyniveneti Folium (Luobumaye) and milk-derived peptides, which all served as ingredients for health food in china (http://samr.cfda.gov.cn/WS01/CL1160/76528.html). The most important active substances of LPS are gastrodin (0.37% *W*/W), flavones (2.2% W/W) and peptides and these ingredients may influence several risk factors for cardiovascular disease. For example, Gastrodia elata Bl. extract suppresses TNF-α-induced vascular inflammatory process and matrix metalloproteinase activity in endothelial cells [18, 19]. The extract of *Eucommia ulmoides* Oliver exhibits antihyperlipidemic properties by suppressing hepatic cholesterol and fatty acid biosynthesis [20] and iridoid and catechol derivatives from *Eucommia ulmoides* Oliver exert beneficial effects on inflammation regulation [21]. In addition, milk-derived bioactive peptides [22], the extracts of *Eucommia ulmoides* Oliver [23] and Apocyniveneti Folium [24] have been reported to possess anti-oxidant activities. Therefore, we determined whether this formula has a beneficial effect on atherosclerotic progression.

## Methods

### Animals

Twenty male ApoE^−/−^ mice on a C57BL/6 J background and 10 male wild-type (C57BL/6 J) mice were obtained from Vital River Laboratory Animal Technology Co., Ltd. (Beijing, China) at aged 7 weeks. The mice were housed individually in climate-controlled rooms (22 ± 1 °C) under diurnal conditions (light–dark: 08:00–20:00) with ad libitum access to tap water and standard laboratory rodent chow. After acclimatization for 7 days, the wild-type mice were fed a low fat diet (10 kcal% fat, 0% cholesterol, Research Diets D12102C, USA) and assigned as control group (CON, *n* = 10). The ApoE^−/−^ mice were fed high fat rodent diet (40 kcal% fat, 1.25% cholesterol, Research Diets D12108C, USA) and randomly assigned to two groups: high fat diet (HFD) group (*n* = 10) and Lipingshu capsule group (n = 10) whose mice received 0.9 g/kg.BW LPS (Infinite Co., LTD. China) orally by a gavage daily for 10 weeks. The animals were cared for in accordance with *the Guiding Principles in the Care and Use of Animals*.

### Lipids analysis

For the measurement of blood lipids, the animals were fasted for 12 h. Blood was collected from animals under deep anesthesia with isoflurane (1.5%) via the retro-orbital venous plexus. Serum obtained by centrifuging at 1500 g (10 min, 4 °C) was stored at − 80 °C. The serum levels of TG, TC, LDL-C and high-density lipoprotein cholesterol (HDL-C) were measured by Hitachi 7020 full-automatic biochemical analyzer (Japan) with commercial kits (Wako, Japan). Serum oxLDL concentrations were analyzed with ELISA kit (Cloud-Clone, Houston, TX).

### Atherosclerosis

The aorta was examined by stereo microscope, isolated and stripped of any external fatty deposits, then longitudinally opened and stained with Oil Red O (sigma, St. Louis, MO). The images are captured with a photo scanner (EPSON GT-X980). The total aortic surface area and Oil Red O-stained area were measured using Image-Pro Plus 6.0 (Media Cybernetics, Rockville, MD, USA).

After embedded with 4% paraformaldehyde, the aortic roots were consecutive sections (10 um) from aortic sinus to aortic arch. Five sections taken at 80 um intervals were examined under a light microscope (Olympus BX61) after staining with hematoxylin-eosine (H&E). The lesion size was determined with Image-Pro Plus 6.0 (Media Cybernetics, Rockville, MD, USA).

### Multiple cytokine measurements

The serum cytokines levels were measured with ProcartaPlex Mouse Cytokine & Chemokine Convenience Panel 1A (36-plex, Thermo Fisher Scientific, Waltham, MA, USA) from eBioscience following the manufacturer’s instructions.

### Statistical analyses

Results are presented as mean ± SEM. Statistical analysis was performed based on t-test and one-way ANOVA using SPSS 13.0 statistical software (SPSS Inc., Chicago, IL). The difference was considered significant when *p* < 0.05.

## Results

### Effects of LPS on atherosclerotic plaques

**Fig. 1:**
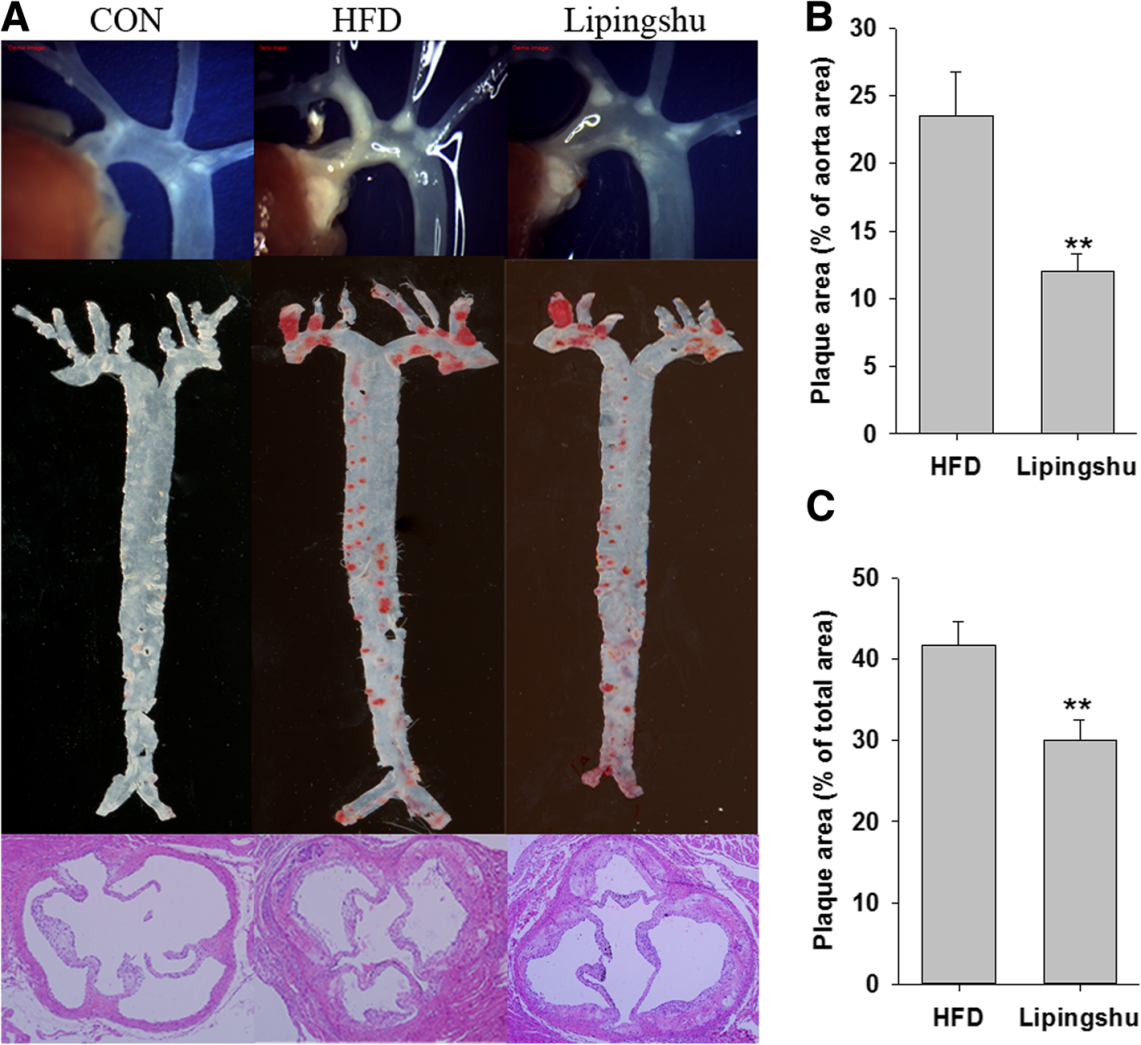
Effects of Lipingshu capsule on atherosclerotic lesions. **a**. Representative photographs of aortic lesions (top), images of Oil Red O-stained (middle), and HE-stained aortic root lesions (bottom). **b**. Areas of the aortic lesion were expressed as the percentage of aorta areas. **c**. Areas of the aortic sinus lesion were expressed as the percentage of total areas. Bars represent mean ± SEM (*n* = 10 animals/group). ** *p* < 0.01 compared to the HFD group

As shown in Fig. 1a, there was no obvious atherogenesis observed by stereo microscope in CON animals. However, ApoE^−/−^ mice fed a HFD had severe aortic lesions. Unanimously, Oil Red O-stained aortas as well as H&E--stained aortic roots both showed pronounced aortic atherosclerotic plaque development in HFD group. The development of aortic lesions was regressed with LPS treatment. The quantitative analysis (Fig. 1b and c) revealed that the plaque areas of the total aorta and aortic root significantly declined in HFD + LPS group.

### Effects of LPS on serum lipid profile

**Fig. 2:**
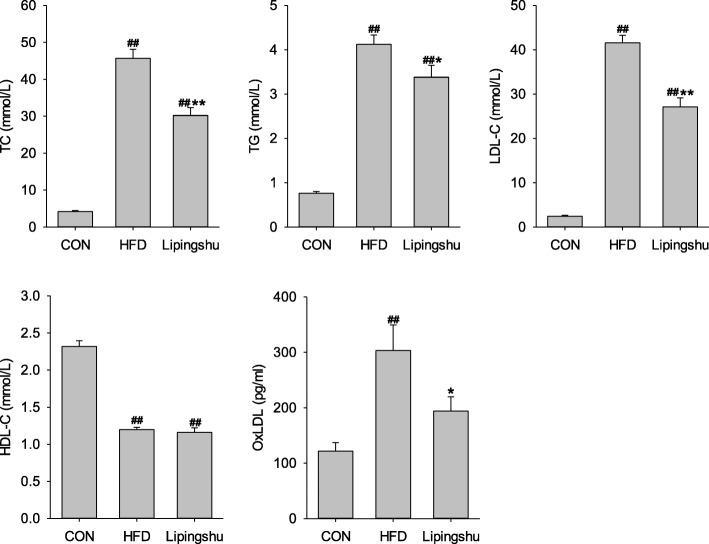
Effects of Lipingshu capsule on the serum lipid profiles. Bars represent mean ± SEM (*n* = 10). ## *p* < 0.01 compared to the CON group, * *p* < 0.05 and ** *p* < 0.01 compared to the HFD group

ApoE^−/−^ mice receiving the HFD considerably heightened the serum levels of TG, TC and LDL-C but slashed the HDL-C level when compared with the control animals (Fig. 2). LPS supplement substantially reduced the contents of serum TG, TC and LDL-C but did not significantly affect HDL-C level. OxLDL levels were remarkably higher in HFD-fed ApoE^−/−^ mice compared with those of control mice, but LPS reduced serum oxLDL concentration significantly.

### Effects of LPS cytokine and chemokine levels

**Fig. 3:**
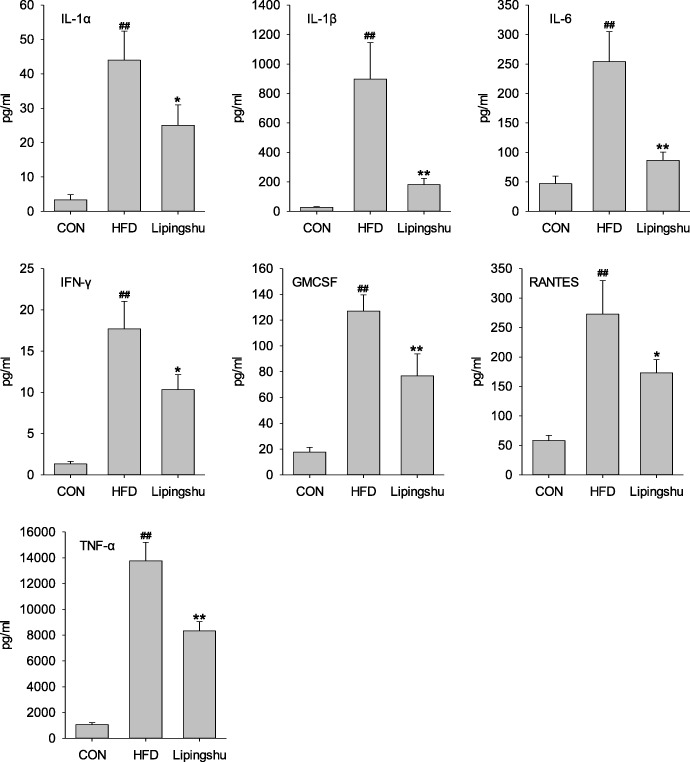
Effects of Lipingshu capsule on serum cytokine and chemokine levels. Bars represent mean ± SEM (*n* = 10). ## *p* < 0.01 compared to the CON group, * *p* < 0.05 and ** *p* < 0.01 compared to the HFD group

To ascertain the systemic effects of LPS on chronic inflammatory response, multi-cytokine analysis was performed to test the levels of serum inflammatory cytokines and chemokines. ApoE^−/−^ mice receiving the HFD had remarkably greater contents of interleukin-1alpha (IL-1α), IL-1β, IL-6, interferon γ (IFN-γ), Granulocyte-Macrophage Colony Stimulating Factor (GMCSF), regulated on activation, normal T cell expressed and secreted (RANTES), and tumour necrosis factor alpha (TNF-α) than control animals (Fig. 3), and these inflammatory cytokines declined remarkably when treated with LPS. The remaining chemokines and cytokines levels were unaffected by LPS (data not shown).

## Discussion

AS is the leading causes of CVD which cause most mortality and morbidity worldwide. Although some TCM herbal formula are valuable resources and therapeutic strategies for AS, the identification of their mechanism of action is a major challenge. The present study revealed that LPS, an innovative formula can be used in health food, possesses protective anti-atherosclerotic effects by improving serum lipids and inflammation.

HFD actually contributes to the development of AS in ApoE^−/−^ mice. In the present study, HFD remarkably promote plaque formation in aortas as well as aortic sinus in atherosclerotic animals. LPS treatment abated atherosclerotic lesions, which was demonstrated by a dramatic diminution in atherosclerotic plaque formation. Hyperlipidemia is a major cause to accelerate AS progression of [25]. It has been widely demonstrated that raised TC and LDL-C contents are atherogenic [26, 27]. The oxidative modification of LDL-C and posterior foam cell formation from macrophage are the hallmarks and pivotal events in the pathogenesis of AS [28]. HDL is believed to possess cardioprotective properties [26, 29] by reversing cholesterol transport, protecting the vascular endothelium, impeding oxidative changes in LDL and exerting anti-inflammatory effects [30, 31]. Therefore, improvement of blood lipid profiles (including increasing HDL-C, reducing TC, LDL-C and TG, and demoting oxLDL generation) represents the most effective prevention strategy for AS. Some TCM herbals have been reported to prevent the lesion development of AS by regulating lipid profiles and blocking LDL oxidation [13, 32]. In the present study, HFD increased serum TG, TC and LDL-C levels and decreased HDL-C level. LPS supplement markedly decreased TG, TC and LDL-C but did not engender HDL-C change in ApoE^−/−^ mice, which leading to the decline in TC/HDL-C as well as LDL-C/HDL-C ratios [33] and thus underpinning the atherosclerotic protection. Extenuating raised ox-LDL levels in HFD-fed atherogenic animals suggested that inhibiting LDL oxidation is another therapeutic approach to AS for LPS.

Numerous basic research have established a prominent role for inflammation in the development of AS [12, 34] and now AS has been established as a chronic inflammatory disease [12]. In AS, the pernicious inflammatory response in atherosclerotic artery increases inflammatory cytokines and other acute-phase reactants contents in blood [34]. On the other hand, a specified group of circulating cytokines, such as TNF-α, IL-1α, IL-1β, IL-2 and IFN-γ, participates in initiating and sustaining full-blown AS [35]. So anti-inflammation is the new therapeutic option for treating AS [36]. In line with previous report [37], HFD induced AS progression was accompanied by marked increment of the pro-atherogenic cytokines, and LPS intervention repressed the excessive production of the inflammation cytokines (such as IL-1α, IL-1β, IL-6, IFN-γ, GMCSF, RANTES and TNF-α). Therefore, LPS may substantially prevent AS by its anti- inflammation activity.

## Conclusions

In conclusion, LPS reduces atherosclerotic plaque burden and thus improves AS by lipid profiles regulation and anti-inflammation. Since all the ingredients of the formula are available in health food stores, LPS may act as a functional food to inhibit AS development and provide effective protection for CVD.

## Abbreviations

ANOVA: Analysis of variance
AS: Atherosclerosis
CVD: Cardiovascular diseases
GMCSF: Granulocyte-Macrophage Colony Stimulating Factor
HDL-C: high-density lipoprotein cholesterol
HFD: High fat diet
IFN-γ: Interferon γ
IL: Interleukin
LDL-C: Low density lipoprotein cholesterol
oxLDL: LDL Oxidative modification
PS: Lipingshu capsule
RANTES: Regulated on activation, normal T cell expressed and secreted
TC: Total cholesterol
TCM: Traditional Chinese Medicine
TG: Triglyceride
TNF-α: Tumour necrosis factor alpha
